# Evaluation of the Efficiency of Detection and Capture of Manganese in Aqueous Solutions of FeCeO_x_ Nanocomposites Doped with Nb_2_O_5_

**DOI:** 10.3390/s20174851

**Published:** 2020-08-27

**Authors:** Artem Kozlovskiy, Kamila Egizbek, Maxim V. Zdorovets, Milana Ibragimova, Alena Shumskaya, Alexandr A. Rogachev, Zhanna V. Ignatovich, Kayrat Kadyrzhanov

**Affiliations:** 1 Engineering Profile Laboratory, L.N.Gumilyov Eurasian National University, Satpaev str. 5, Nur-Sultan 010008, Kazakhstan; kemelin@mail.ru (K.E.); mzdorovets@gmail.com (M.V.Z.); i.m.13@bk.ru (M.I.); kayrat.kadyrzhanov@mail.ru (K.K.); 2 Laboratory of Solid State Physics, The Institute of Nuclear Physics, Ibrag and ov str. 1, Almaty 050032, Kazakhstan; 3 Department of Intelligent Information Technologies, Ural Federal University, Mira str. 19, 620002 Ekaterinburg, Russia; 4 Optical anisotropic films laboratory, Institute of Chemistry of New Materials of the National Academy of Sciences of Belarus, 22007 Minsk, Belarus; shumska.alena@gmail.com (A.S.); rogachev78@mail.ru (A.A.R.); ignatovich@ichnm.by (Z.V.I.)

**Keywords:** nanocomposites, chemical deposition, water purification, doping, FeNbO_4_

## Abstract

The main purpose of this work is to study the effectiveness of using FeCeO_x_ nanocomposites doped with Nb_2_O_5_ for the purification of aqueous solutions from manganese. X-ray diffraction, energy–dispersive analysis, scanning electron microscopy, vibrational magnetic spectroscopy, and mössbauer spectroscopy were used as research methods. It is shown that an increase in the dopant concentration leads to the transformation of the shape of nanoparticles from spherical to cubic and rhombic, followed by an increase in the size of the nanoparticles. The spherical shape of the nanoparticles is characteristic of a structure consisting of a mixture of two phases of hematite (Fe_2_O_3_) and cerium oxide CeO_2_. The cubic shape of nanoparticles is typical for spinel-type FeNbO_4_ structures, the phase contribution of which increases with increasing dopant concentration. It is shown that doping leads not only to a decrease in the concentration of manganese in model solutions, but also to an increase in the efficiency of adsorption from 11% to 75%.

## 1. Introduction

In recent years, special attention has been paid to nanocomposites based on metal oxides or ferrites, interest in which is due to the high potential for their use as catalysts, the basis for carriers of drugs, sensors and adsorbents of pollutants, and waste water treatment [1,2,3,4,5]. The wide range of their application is based on the possibility of obtaining nanostructured composites with large specific surface area, functional groups, and resistance to degradation [6,7]. Magnetic characteristics such as coercivity and saturation magnetization in turn allow the use of nanoparticles for purification of aqueous media by adsorption of contaminants on its surface and subsequent removal from solution by magnetic separation [8,9,10].

Among the variety of metal oxide nanocomposites and nanoparticles FeCeO_x_ structures of the type have great potential in photocatalysis, biomedicine, purification of aqueous media, etc. [7,8,9,10,11,12]. Methods for preparing FeCeO_x_ nanostructures are quite numerous, and in most of them multiphase amorphous-like structures are obtained, which makes their own adjustments for their application [13,14,15]. Additionally, the use of iron-containing nanoparticles or composites based on them is accompanied by a rapid rate of degradation of the crystal structure as a result of the oxidation of iron and its subsequent decay. One way to increase resistance to degradation, as well as changes in functional properties and surface is to dope nanocomposites FeCeO_x_ various elements or compounds, which significantly increases their productivity, as well as structural and morphological properties [16,17,18,19,20].

Due to the aforesaid, the purpose of this work is consideration of a possibility of increase in efficiency of filtration and cleaning of water environments by means of nanocomposites of FeCeO_x_ doped by oxide of niobium (Nb_2_O_5_). The choice as a dopant is Nb_2_O_5_ due to its wide use as a doping element of various composite materials in order to increase photocatalytic activity, due to the small width of the forbidden zone, good resistance to degradation, as well as the possibility of obtaining spinel stable phases [21,22,23,24,25]. The relevance of the study is to assess the potential use of nanocomposites for the purification of aqueous media from pollutants such as manganese, which is one of the harmful metals concentrated in wastewater. The possibility of adsorption of manganese on the surface of nanocomposites and their subsequent capture by magnets can be further used on a semi-industrial or industrial scale.

## 2. Experimental Part

The synthesis of nanocomposites was carried out in two stages, including the chemical deposition of FeCeO_x_ (which is a mixture of two phases Fe_2_O_3_ and CeO_2_) [26] and subsequent mechanochemical mixing of amorphous-like FeCeO_x_ nanoparticles with Nb_2_O_5_ nanoparticles in the set stoichiometric proportions and thermal annealing in the air atmosphere at a temperature of 1000 °C within 8 h.

The samples were cooled down together with a muffle furnace (Naberthetm, Bremen, Germany) for 24 h. The choice of the annealing temperature is due to phase transformations in FeCeO_х_ nanoparticles with the formation of two stable phases Fe_2_O_3_ and CeO_2_. Earlier in [26], we examined in detail the phase transformations of FeCeO_х_ nanoparticles as a result of thermal annealing in the temperature range from 400 to 800 °C, according to which, when the annealing temperature is increased above 600 °C, the structure of nanoparticles is a mixture of two phases Fe_2_O_3_ and CeO_2_. In this case, an increase in temperature leads to an increase in the degree of structural ordering and a decrease in distortions and deformations of the crystal lattice. However, experimental work with an increase in the annealing temperature above 1000 °C led to the formation of large agglomerates of micron-sized particles that are not suitable for research.

The study of changes in the shape and size of nanoparticles as a result of changes in the concentration of the dopant was carried out by analyzing images obtained using a scanning electron microscope TM3030 (Hitachi Ltd., Chiyoda, Tokyo, Japan). The study of the elemental composition and its change were carried out using the method of energy dispersive analysis performed on the attachment Bruker XFlash MIN SVE microanalysis system at voltage of 15 kV.

The phase composition and the dynamics of its change as a result of doping were determined on the basis of the obtained diffractograms recorded on a D8 Advance ECO powder diffractometer (Bruker), in the Bragg–Brentano geometry in the angle range 2θ = 20–80° with a step of 0.03°, radiation Cu-Kλ = 1.54 Å.

The determination of magnetic characteristics was carried out using the vibration magnetic spectroscopy method performed on the “Liquid Helium Free High Field Measurement System”, “Cryogenic LTD”.

The study of ultrafine magnetic parameters was carried out using the method of Mössbauer spectroscopy on the MS1104Em Mössbauer spectrometer (Ritverc, Chernogolovka, Russia) with a ^57^Co nuclei in the Rh matrix source. The calibration of the spectra obtained at room temperature was carried out using α-Fe absorber (Ritverc, Chernogolovka, Russia).

The study of the prospects of using the synthesized nanoparticles before and after doping as a basis for the purification of aqueous media from manganese by adsorption on the surface of nanoparticles and subsequent filtration was carried out using model aqueous solutions with a diluted manganese concentration of 22 mg/dm^3^. During the experiment, the solution was stirred to avoid the formation of a precipitate at the bottom of the beaker. Determination of the concentration of manganese before and after adsorption was carried out by determining the optical density in the wavelength range of 440–460 nm using the analyzer “Fluorat-02”. The concentration of nanoparticles was 0.001 g, the adsorption time was 5 h. After the end of the time, the nanoparticles were captured by the magnet and removed from the model medium.

## 3. Results and Discussion

**Figure 1:**
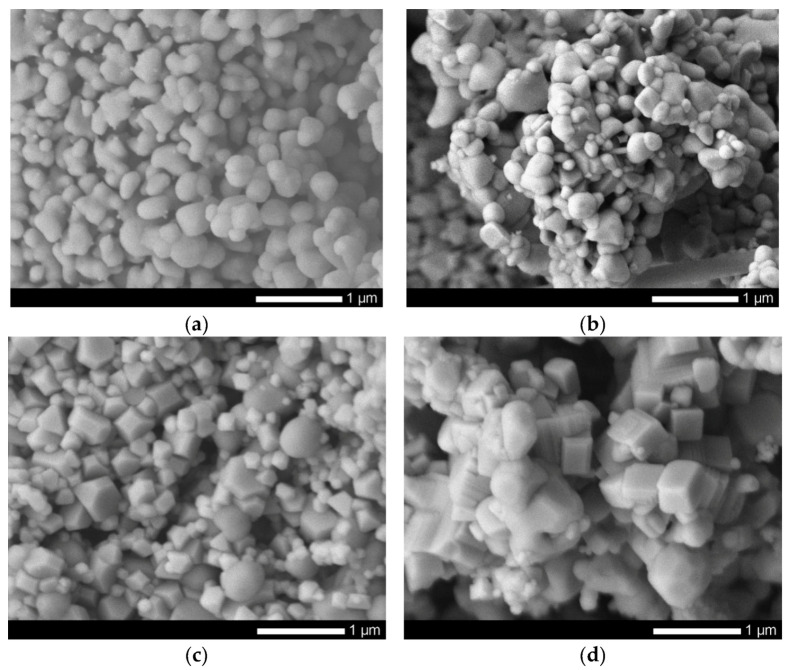
SEM images of synthesized nanoparticles: (**a**) FeCeO_x_ nanoparticles; (**b**) 0.9FeCeO_x_ + 0.1Nb_2_O_5_ nanoparticles; (**c**) 0.8FeCeO_x_ + 0.2 Nb_2_O_5_ nanoparticles; and (**d**) 0.7FeCeO_x_ + 0.3 Nb_2_O_5_ nanoparticles.

**Figure 2:**
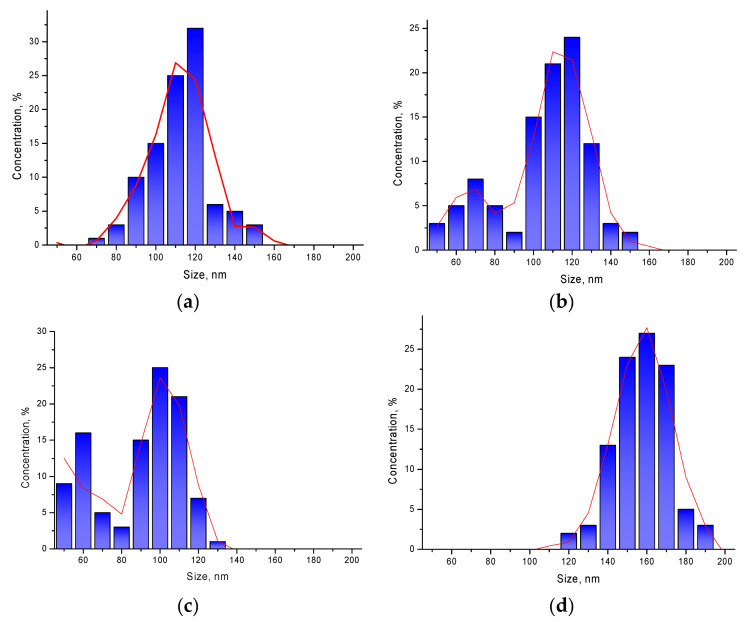
Diagrams of nanoparticle size distribution depending on the dopant concentration: (**a**) FeCeO_x_ nanoparticles; (**b**) 0.9FeCeO_x_ + 0.1Nb_2_O_5_ nanoparticles; (**c**) 0.8FeCeO_x_ + 0.2 Nb_2_O_5_ nanoparticles; and (**d**) 0.7FeCeO_x_+ 0.3 Nb_2_O_5_ nanoparticles.

Figure 1 shows images obtained using the method of scanning electron microscopy of synthesized nanoparticles before and after modification by doping. In the initial state, FeCeO_3_ nanoparticles are spherical particles, the average size of which, according to the distribution diagram (see Figure 2a), varies from 110–120 nm.

**Figure 3:**
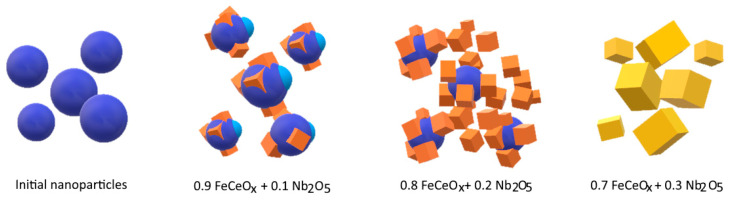
Schematic representation of the transformation of the geometry of the synthesized nanoparticles as a result of an increase in the concentration of the dopant Nb_2_O_5_.

Doping of Nb_2_O_5_ with increasing concentration leads to changes not only in the size of nanoparticles, but also in the shape. A schematic representation of the resizing is shown in Figure 3.

Initially, spherical nanoparticles undergo transformation in several stages, depending on the concentration of the dopant. In the case when the concentration is 0.1–0.2 of the total mass of mixed nanoparticles, spherical nanoparticles first lose their shape due to fouling by growths and cubic or diamond-shaped inclusions, which is also reflected in changes in the size of nanoparticles (see Figure 2b,c) by the appearance of a second maximum in the region 60–70 nm. An increase in the concentration of the dopant to 0.3 leads to a complete transformation of nanoparticles from a spherical to a cubic or rhomboid shape, accompanied by an increase in the size of nanoparticles to 150–170 nm (see Figure 2d).

The Table 1 shows the elemental analysis data of the studied nanoparticles depending on the concentration of the dopant, as well as the specific surface area and the ratio of spherical and cubic nanoparticles.

As can be seen from the presented data, an increase in the concentration of the dopant Nb_2_O_5_ leads to the displacement of cerium atoms from the structure, while the iron content remains practically unchanged. Moreover, a change in the shape and size of nanoparticles leads to an increase in the specific surface area. However, for the case when the concentration of the dopant Nb_2_O_5_ was 0.3 of the total mass, which is characterized by the transformation of nanoparticles from a spherical to a cubic shape, a decrease in the specific surface area is observed. Furthermore, a change in the concentration of the dopant Nb_2_O_5_ leads to a change in the ratio of the types of particles with an almost complete predominance of the cubic and rhombic forms at a concentration of the dopant Nb_2_O_5_ of 0.3 of the total mass.

**Figure 4:**
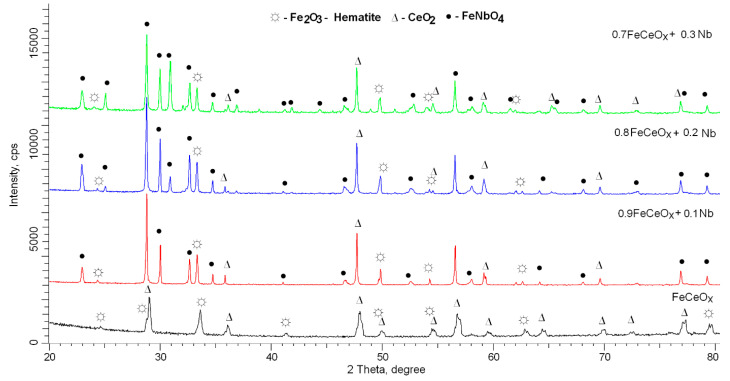
X-ray diffraction pattern of the studied samples FeCeO_x_ nanoparticles doped Nb_2_O_5_ with different concentration.

**Figure 5:**
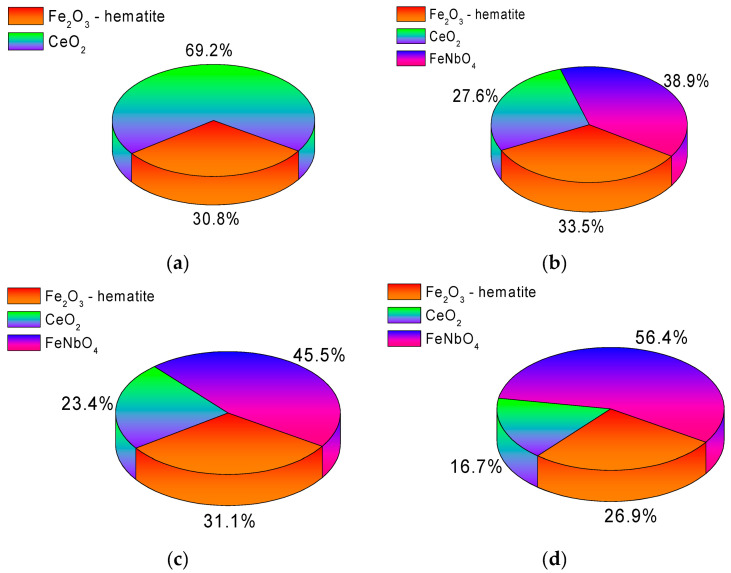
Phase analysis data obtained by analyzing X-ray diffractograms: (**a**) FeCeO_x_ nanoparticles; (**b**) 0.9FeCeO_x_ + 0.1Nb_2_O_5_ nanoparticles; (**c**) 0.8FeCeO_x_ + 0.2 Nb_2_O_5_ nanoparticles; and (**d**) 0.7FeCeO_x_ + 0.3 Nb_2_O_5_ nanoparticles.

The assessment of the phase composition of the studied nanoparticles depending on the concentration of the dopant Nb_2_O_5_ is presented in Figure 4 (X-ray diffraction data) and Figure 5 (phase composition data). The general view of the diffraction patterns obtained using the shooting method in the Bragg–Brentano geometry indicates the polycrystallinity of the synthesized nanoparticles, as well as the presence of structural distortions arising in the process of grinding and subsequent thermal annealing.

The different shapes of diffraction reflections for the initial sample indicate the presence in the structure of two phases characteristic of hematite (Fe_2_O_3_) and cerium oxide CeO_2_, the phase ratio of which is approximately 1:2. In the case of doped nanoparticles, a change in the diffraction patterns is observed associated with the appearance of new additional peaks at 2θ = 24.2°, 25.7°, 28.1°, 30.2°, 31.8°, and 32.9°, characteristic of the monoclinic phase of FeNbO_4_-spinel type, whose contribution increases with increasing concentration dopant. In this case, the peaks characteristic of the Fe_2_O_3_ and CeO_2_ phases are also present in the diffractograms; however, their intensities decrease with an increase in the dopant concentration. The appearance of a new FeNbO_4_ phase in the structure of nanoparticles indicates a phase transformation that occurs during grinding and subsequent annealing. The diagrams of the phase ratios in the composition of nanoparticles presented in Figure 5 were obtained by full-profile analysis of X-ray diffractograms with the subsequent calculation of the contributions according to formula (1) [27]:

(1) $$V_{\mathrm{admixture}}=\frac{RI_{\mathrm{phase}}}{I_{\mathrm{admixture}}+RI_{phase}},$$

*I*_phase_ is the value of the integral intensity of the main phase, *I*_admixture_ is the value of the integral intensities of impurity phases, the structure factor is *R* = 1.45.

**Figure 6:**
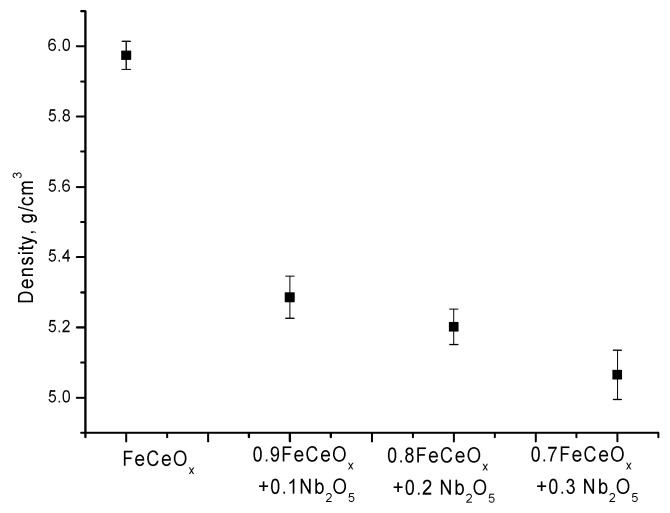
Graph of changes in the density of nanoparticles depending on the concentration of the dopant.

According to the data presented in the diffraction patterns, an increase in the concentration of the dopant Nb_2_O_5_ leads to the displacement of the CeO_2_ phase from the structure of nanoparticles, as well as to the dominance of the FeNbO_4_ phase. In this case, a change in the phase composition leads to a change in the density of nanoparticles, the data of which are shown in Figure 6.

**Figure 7:**
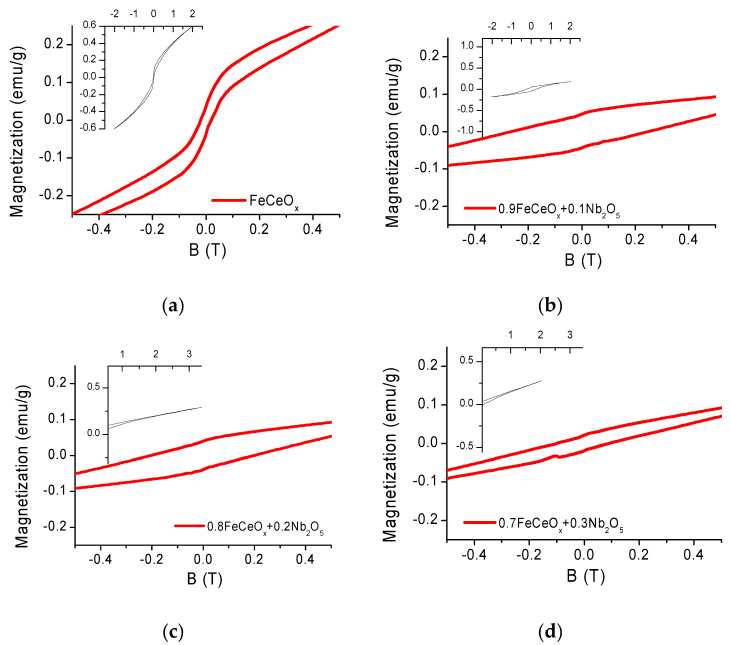
Hysteresis loops of the studied nanoparticles: (**a**) FeCeO_x_ nanoparticles; (**b**) 0.9FeCeO_x_ + 0.1Nb_2_O_5_ nanoparticles; (**c**) 0.8FeCeO_x_ + 0.2 Nb_2_O_5_ nanoparticles; and (**d**) 0.7FeCeO_x_ + 0.3 Nb_2_O_5_ nanoparticles.

The magnetic characteristics of nanoparticles depending on the concentration of the dopant were studied using a vibrating magnetometer, hysteresis loops are shown in Figure 7, the results of calculating the magnetic characteristics are presented in Table 2.

In the initial state, nanoparticles have a significant coercivity value Hc = 200, Oe and small values of specific magnetic parameters Mr = 0.0375, emu/g, Ms = 0.26, emu/g.

The literature provides various values of magnetic parameters of the composite FeCeO_x_ [27,28,29], which is associated with the particle size of iron oxide and its fraction in the composites. With sizes up to tens of nanometers, particles have superparamagnetic characteristics, with large sizes-ferromagnetic properties. The samples in question have an average size of 120 nm and have ferromagnetic properties, and low values of specific parameters are explained by the high content of non-magnetic phase in the composite.

When Nb_2_O_5_ is introduced into the reaction mixture and the FeNbO_4_ phase is formed in the structure of nanoparticles, there is a sharp change in the magnetic parameters, the connectivity increases 13 times for 0.9FeCeO_x_ + 0.1Nb_2_O_5_ nanoparticles and 10 times for 0.8FeCeO_x_ + 0.2Nb_2_O_5_ nanoparticles, the quadraticity of the hysteresis loops changes. The observed change is associated with surface magnetic effects occurring on FeCeO_x_ particles, apparently, by the pinning of magnetic moments due to the formation of a new phase. However, a significant change in the phase composition in the 0.7FeCeO_x_ + 0.3Nb_2_O_5_ nanoparticles sample and the formation of a new shape of structures leads to a weakening of the surface effects and a halving of the coercivity.

**Figure 8:**
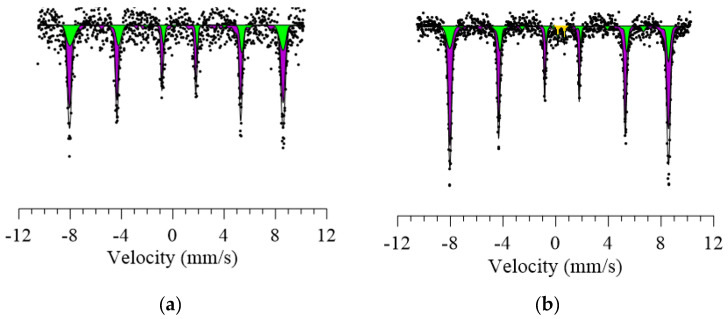

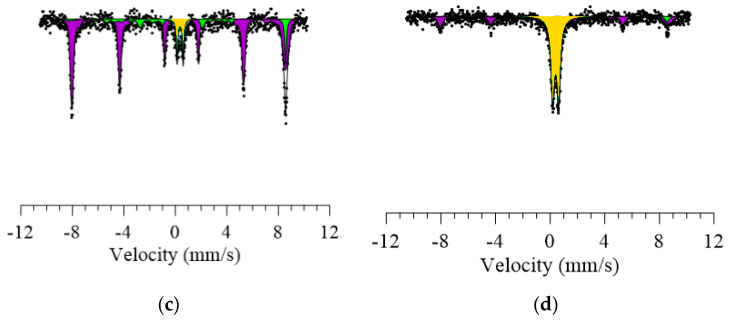
Mössbauer spectra of nanoparticles: (**a**) FeCeO_x_ nanoparticles; (**b**) 0.9FeCeO_x_ + 0.1Nb_2_O_5_ nanoparticles; (**c**) 0.8FeCeO_x_ + 0.2 Nb_2_O_5_ nanoparticles; and (**d**) 0.7FeCeO_x_ + 0.3 Nb_2_O_5_ nanoparticles.

The estimation of the hyperfine magnetic parameters of the synthesized nanoparticles, as well as the effect of doping on the degree of magnetic disordering, was carried out by analyzing the Mössbauer spectra obtained at room temperature. The general view of the spectrum of nanoparticles in the initial state is characterized by the presence of two sextets of ultrafine magnetic fields of 515–516 kOe, which are characteristic of two types of ferromagnetic iron atoms (see Figure 8). The values of the hyperfine fields and also isomeric shifts for the initial nanoparticles are characteristic of structures such as hematite Fe_2_O_3_ with a partially disordered structure. Disordering and deviation of the hyperfine fields from the reference values of hematite is due to the presence of the CeO_2_ phase in the structure of nanoparticles. Additionally, the influence of the CeO_2_ phase on the degree of magnetic and structural ordering is evidenced by the ratio of the contributions of the partial spectra of two sextets 2.11:0.89, which is characteristic of the violation of stoichiometry at various levels of population in the A- and B-sublattices.

**Figure 9:**
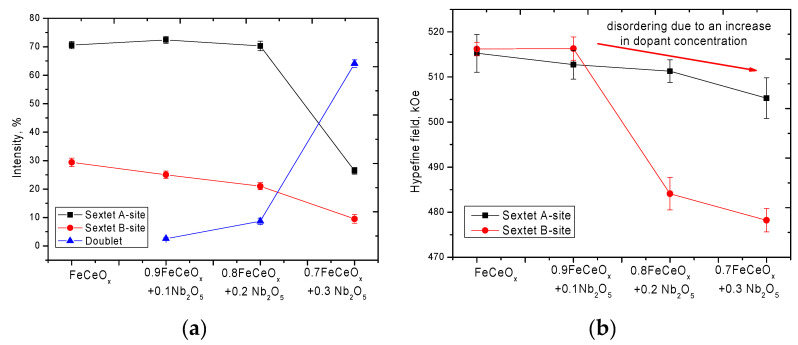
Changes in the hyperfine parameters of magnetic characteristics obtained on the basis of the analysis of Mössbauer data: (**a**) data of changes of contribution intensities of partial spectra depending on concentration of dopant Nb_2_O_5_; (**b**) data on changes in the magnitude of hyperfine magnetic fields in the A- and B- sublattices.

Figure 9 shows the data on the variation of the intensities of the contributions of the partial spectra depending on the concentration of the dopant Nb_2_O_5_, as well as the magnitude of the hyperfine magnetic fields in the A- and B- sublattices obtained from the analysis of the Mössbauer spectra.

For microparticles doped with Nb_2_O_5_ the emergence of a doublet and also decrease in the intensities of two sextets with the subsequent complete dominance of a doublet in the structure of the spectrum of 0.7FeCeO_x_ + 0.3 Nb_2_O_5_ nanoparticles is observed. The appearance of a quadrupole paramagnetic doublet and the subsequent increase in its contribution is due to the doping effect and partial replacement of iron atoms with niobium with the subsequent formation of a spinel structure. At the same time, the decrease in the value of ultra-thin magnetic fields in the case of an increase in the concentration of dopant Nb_2_O_5_ may be due to the presence of a phase FeNbO_4_ in the structure, which is characterized by the spinel structure.

**Figure 10:**
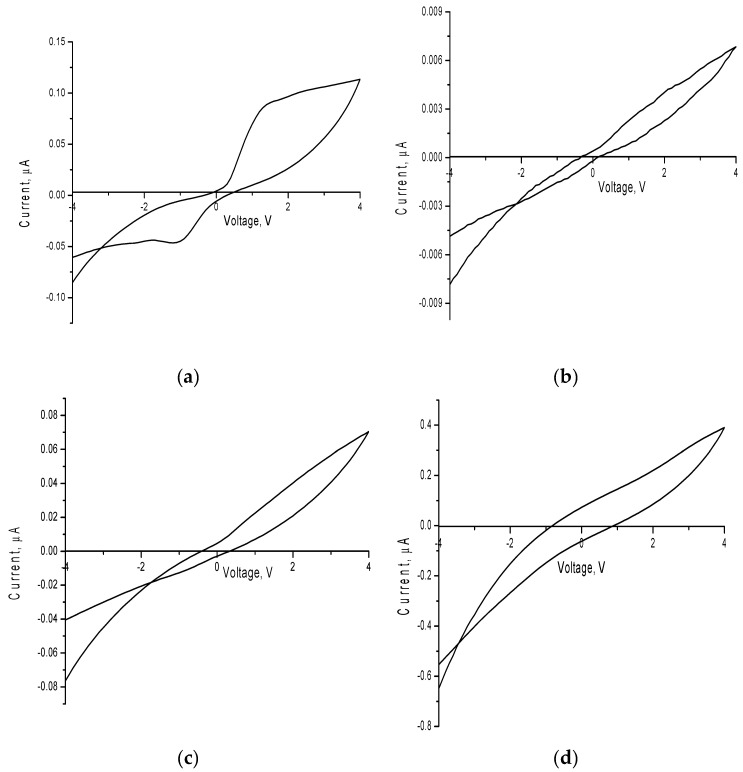
Graphs of cyclic voltammograms of the studied nanoparticles: (**a**) FeCeO_x_ nanoparticles; **b**) 0.9FeCeO_x_ + 0.1Nb_2_O_5_ nanoparticles; (**c**) 0.8FeCeO_x_ + 0.2 Nb_2_O_5_ nanoparticles; and (**d**) 0.7FeCeO_x_ + 0.3 Nb_2_O_5_ nanoparticles.

Figure 10 shows the data on changes in the cyclic current-voltage characteristics of the studied nanoparticles depending on the concentration of the dopant. For the initial nanoparticles, the nature of the change in the cyclic volt-amperogram has a nonlinear non-ohmic character, characterized by a region from 0.2 to 1.5 V with a sharp increase in conductivity, characteristic of the quadratic dependence of the change in current strength on the applied voltage, and subsequent smooth change in current strength depending on voltage. Furthermore, the presence of an abrupt behavior of a change in the current-voltage characteristic can be due to the presence of two phases in the structure, which create additional obstacles to the ballistic nature of charge transfer.

**Figure 11:**
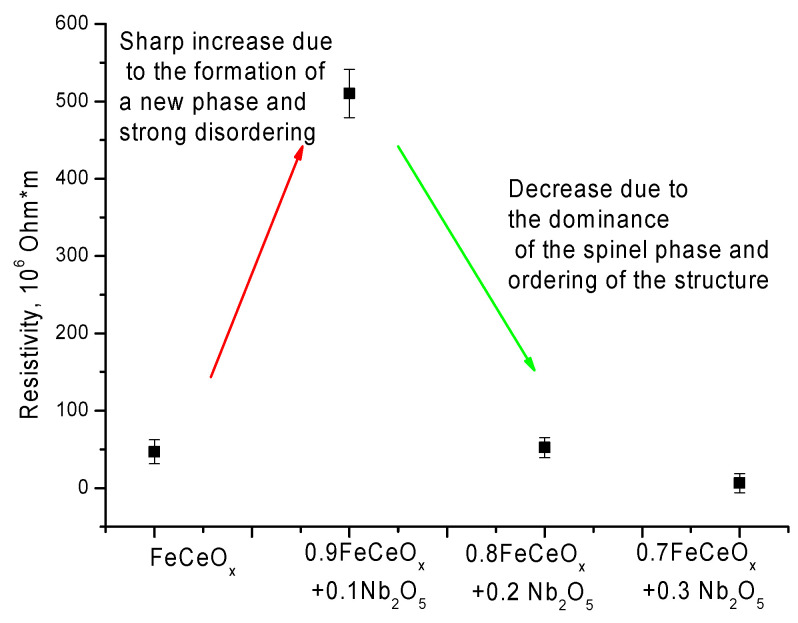
Graph of change of resistance value depending on type of nanoparticles.

For doped nanoparticles, the character of the change in current-voltage characteristics is close to ohmic, without the obvious presence of regions with a quadratic dependence of the change in I on V. In this case, the formation of the FeNbO_4_ phase at low concentrations leads to a sharp increase in the resistivity of nanoparticles (see Figure 11). However, the dominance of the FeNbO_4_ phase, as well as transformation processes in the shape of nanoparticles, lead to structure ordering and a decrease in the resistance value.

**Figure 12:**
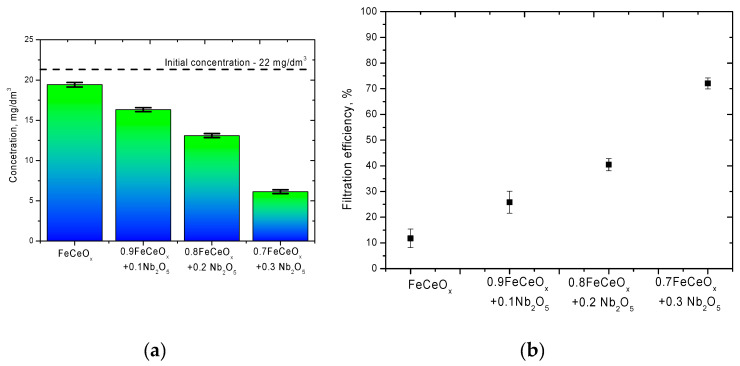
Results of filtration characteristics of aqueous solutions from nanoparticles: (**a**) diagram of manganese concentration change in model solutions after 5 h of filtration; (**b**) graph of efficiency of manganese filtration from model solutions.

Figure 12 shows the results of experiments on the purification of aqueous model solutions using synthesized nanoparticles from manganese by adsorption on the surface of nanoparticles and subsequent decrease in concentration in the model solution. An aqueous solution with a dissolved manganese concentration of 22 mg/dm^3^ was used as a model medium. The time of adsorption by placing 0.001 g of synthesized nanoparticles in a model solution was 5 h. After the end of the time, the nanoparticles were captured by the magnet and removed from the model medium.

**Figure 13:**
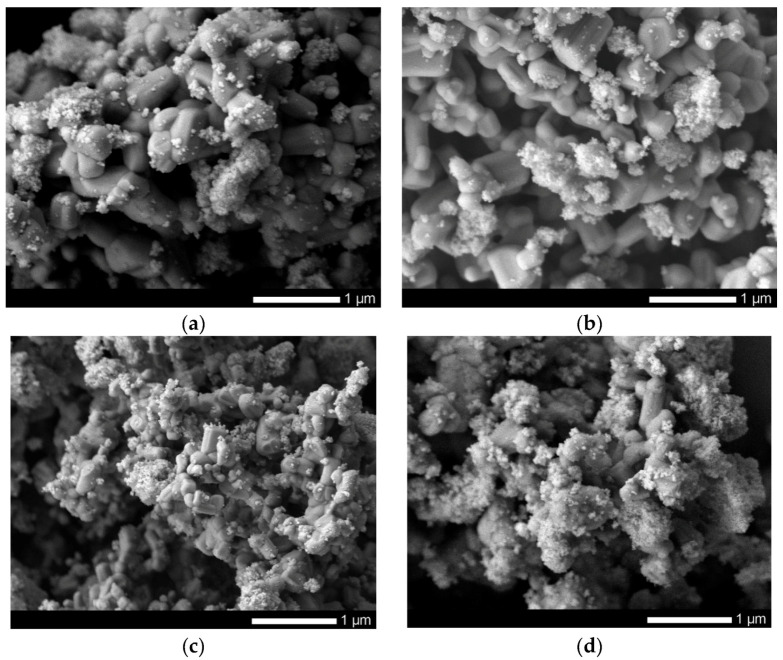
SEM images of synthesized nanoparticles after separation: (**a**) FeCeO_x_ nanoparticles; (**b**) 0.9FeCeO_x_ + 0.1Nb_2_O_5_ nanoparticles; (**c**) 0.8FeCeO_x_ + 0.2 Nb_2_O_5_ nanoparticles; and (**d**) 0.7FeCeO_x_ + 0.3 Nb_2_O_5_ nanoparticles.

According to the presented data, in the case of using the initial nanoparticles, the efficiency of reducing the concentration of manganese in the model solution after filtration was no more than 11%, while for doped nanoparticles, an increase in the concentration of the dopant leads to a sharp increase from 24% to 75% (see Figure 12b). An increase in the adsorption capacity of doped nanoparticles is due to a change in the morphological features of nanoparticles, as well as structural transformations, which more actively interact with the model solution and adsorb manganese on their surface, followed by its deposition on the surface of nanoparticles in the form of build-ups and small particles, which are clearly visible on SEM images nanoparticles after the filtration process (see Figure 13).

**Figure 14:**
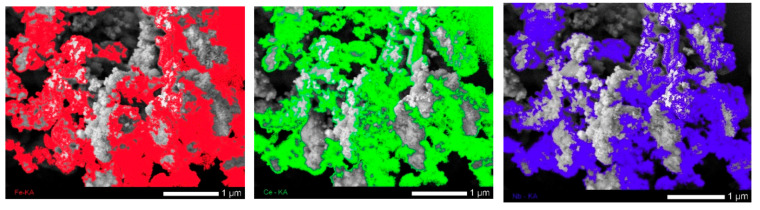

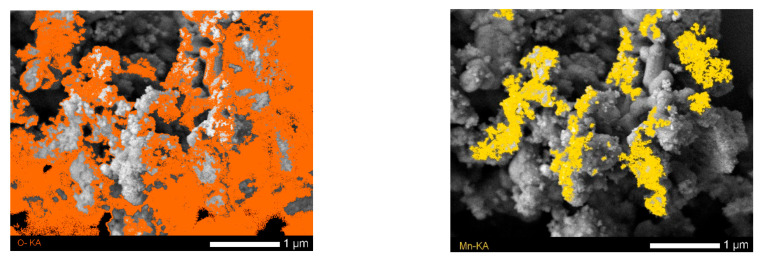
Mapping results for 0.7FeCeO_x_ + 0.3 Nb_2_O_5_ nanoparticles after filtration. (The color in the figure indicates the distribution of elements determined according to the mapping data: red—Fe, green—Ce, blue—Nb, orange—O, and yellow—Mn.)

A direct confirmation of the fact that the formed small growths on the surface of nanoparticles are manganese are the data of the mapping results, which are presented in Figure 14, obtained from the 0.7FeCeO_x_ + 0.3 Nb_2_O_5_ nanoparticles samples.

**Figure 15:**
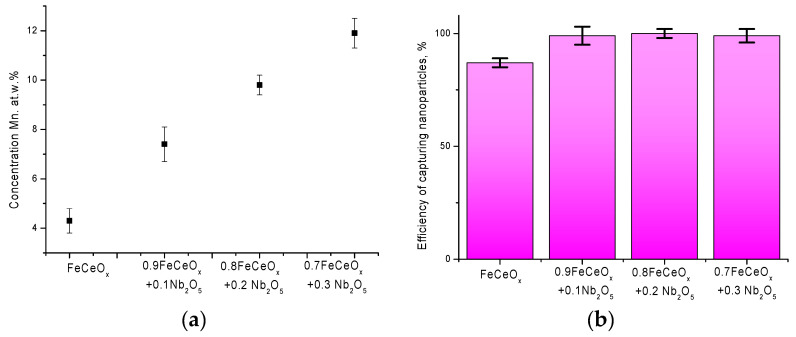
Data of energy dispersion analysis and conservation of mass of nanoparticle samples after filtration: (**a**) graph of changes in the concentration of manganese on the surface of nanoparticles after filtration; (**b**) graph of the efficiency of capturing nanoparticles after filtration.

Figure 15a shows data on changes in the elemental content of manganese on the surface of nanoparticles, which was determined using the method of energy dispersive analysis. According to the data obtained, for the initial nanoparticles, the content of adsorbed manganese on the surface is no more than 4–5 at %, while for doped nanoparticles this value ranges from 7% to 12% depending on the concentration of the dopant.

An important feature of magnetic nanoparticles used for purification of aqueous media or photocatalytic decomposition reactions is the possibility of their complete extraction from solutions after filtration and a cycle of reactions using magnets. Figure 15b shows the data on the efficiency of trapping nanoparticles using magnets after 5 h of filtration, from which it can be seen that the percentage of trapping nanoparticles after filtration is 97–100% for modified nanoparticles, which indicates that the magnetic properties of nanoparticles are preserved after filtration. In this case, the degree of capture of 87% for the initial nanoparticles indicates a partial degradation of nanoparticles during the experiment. So, for example, in work [30] it was shown that after 24 h of staying in an aqueous solution of nanoparticles, their partial degradation is observed due to the formation of paramagnetic inclusions, which can reduce the collection efficiency. In this case, a long stay in the model solution leads to a decrease in the filtration efficiency due to the degradation of nanoparticles. However, in our case, the choice of a filtration time of five hours does not lead to any significant changes in the morphology of nanoparticles, as well as their degradation.

## 4. Conclusions

The paper demonstrates the efficiency of using FeCeO_x_ doping of Nb_2_O_5_ nanoparticles in order not only to change the morphology and phase composition of nanoparticles, but also to increase the adsorption of manganese and increase the degree of purification. Resistance to degradation with an increase in the concentration of the dopant Nb_2_O_5_, as well as an increase in the purification efficiency, is due to the presence of a stable phase of the spinel type FeNbO_4_ in the structure. The high degree of capture efficiency for modified nanoparticles indicates the possibility of their reuse.

Further experiments will be aimed at studying the influence of various factors, such as the acidity of the medium, the concentration of nanoparticles in the model solution on the degree of purification.

## Figures and Tables

**Table 1:** Elemental analysis data, specific surface area, and particle type ratio.

Sample	Fe, at.w.%	Ce, at.w.%	Nb, at.w.%	O, at.w.%	Specific Surface Area, m^2^/g	Ratio of Spherical and Cubic Nanoparticles
**FeCeO_x_ nanoparticles**	14.1 ± 1.1	29.5 ± 2.2	-	56.4 ± 3.4	0.0087	100:0
**0.9FeCeO_x_ + 0.1Nb_2_O_5_ nanoparticles**	13.2 ± 1.5	22.2 ± 2.1	9.2 ± 1.2	55.4 ± 3.1	0.0109	78:22
**0.8FeCeO_x_ + 0.2 Nb_2_O_5_ nanoparticles**	13.6 ± 1.2	13.4 ± 1.3	14.1 ± 1.5	58.9 ± 2.5	0.0126	34:66
**0.7FeCeO_x_ + 0.3 Nb_2_O_5_ nanoparticles**	13.2 ± 1.3	7.8 ± 0.7	19.4 ± 1.2	59.6 ± 2.7	0.0098	3:97

**Table 2:** Magnetic characteristics data.

Sample	Hc, Oe ^1^	Mr, emu/g ^2^	Ms, emu/g ^3^	Mr/Ms ^4^
FeCeO_x_ nanoparticles	200	0.0375	0.26	0.144
0.9FeCeO_x_ + 0.1Nb_2_O_5_ nanoparticles	2586	0.0448	0.195	0.23
0.8FeCeO_x_ + 0.2Nb_2_O_5_ nanoparticles	2035	0.0395	0.375	0.105
0.7FeCeO_x_ + 0.3Nb_2_O_5_ nanoparticles	960	0.0207	0.189	0.11

^1^ H_c_, Oe—coercivity, ^2^ Mr, emu/g—remanent magnetization, ^3^ Ms, emu/g—saturation magnetization, ^4^ Mr/Ms—squareness.

## References

[1] Niu L., Wei T., Li Q., Zhang G., Xian G., Long Z., Ren Z. (2020). Ce-based catalysts used in advanced oxidation processes for organic wastewater treatment: A review. J. Environ. Sci..

[2] Zhang Q., Zhou W., Cui Y., Lyu C., Liu T., Zhang R., Zhang R., Zheng J., Shi Z., Lu C. (2018). Iron triad nanomaterials and their sustainable application in the environment. Environ. Sci. Nano.

[3] Balcaen V., Poelman H., Poelman D., Marina G.B. (2011). Kinetic modeling of the total oxidation of propane over Cu-and Ce-based catalysts. J. Catal..

[4] Zhang N., Zhang G., Chong S., Zhao H., Huang T., Zhu J. (2017). Ultrasonic impregnation of MnO2/CeO2 and its application in catalytic sono-degradation of methyl orange. J. Environ. Manag..

[5] Weia X., Zhuabcd N., Huanga X., Kanga N., Wuabcd P., Dangab Z. (2020). Efficient degradation of sodium diclofenac via heterogeneous Fenton reaction boosted by Pd/Fe@Fe3O4 nanoparticles derived from bio-recovered palladium. J. Environ. Manag..

[6] Khataee A., Gholami P., Kalderis D., Pachatouridou E., Konsolakis M. (2018). Preparation of novel CeO2-biochar nanocomposite for sonocatalytic degradation of a textile dye. Ultrason. Sonochem..

[7] Chong S., Zhang G., Zhang N., Liu Y., Zhu J., Huang T., Fang S. (2016). Preparation of FeCeO x by ultrasonic impregnation method for heterogeneous Fenton degradation of diclofenac. Ultrason. Sonochem..

[8] Huang F., Wang J., Chen W., Wan Y., Wang X., Cai N., Liu J., Yu F. (2018). Synergistic peroxidase-like activity of CeO 2 -coated hollow Fe 3 O 4 nanocomposites as an enzymatic mimic for low detection limit of glucose. J. Taiwan Inst. Chem. Eng..

[9] Mura S., Jiang Y., Vassalini I., Gianoncelli A., Alessandri I., Granozzi G., Calvillo L., Senes N., Enzo S., Innocenzi P. (2018). Graphene Oxide/Iron Oxide Nanocomposites for Water Remediation. ACS Appl. Nano Mater..

[10] Kang Y.-G., Yoon H., Lee C.-S., Kim E.-J., Chang Y.-S. (2019). Advanced oxidation and adsorptive bubble separation of dyes using MnO2-coated Fe3O4 nanocomposite. Water Res..

[11] Wang L., Wang J., Liu X., Chen Y., Cheng H., Wu Y., Peng H., Mab Z. (2020). FeCeOx with improved activity for catalytic reduction of NO with NH3. J. Phys. Chem. Solids.

[12] Khataee A., Hassandoost R., Pouran S.R. (2018). Cerium-substituted magnetite: Fabrication, characterization and sonocatalytic activity assessment. Ultrason. Sonochem..

[13] Zhou H., Zhao Z. (2015). The Preparation, Characterization and Photocatalytic Activity of FexCe1-xO2-n Microstructures. Integr. Ferroelectr..

[14] Liu J., Zhou J., Ding Z., Zhao Z., Xu X., Fang Z. (2017). Ultrasound irritation enhanced heterogeneous activation of peroxymonosulfate with Fe 3 O 4 for degradation of azo dye. Ultrason. Sonochem..

[15] Channei D., Inceesungvorn B., Wetchakun N., Phanichphant S., Nakaruk A., Koshy P., Sorrell C. (2013). Photocatalytic activity under visible light of Fe-doped CeO2 nanoparticles synthesized by flame spray pyrolysis. Ceram. Int..

[16] Zhang H., Zhang M., Hao L., Wang J., Ma Y., Zhang Y., Jiao T., Zhang W., Chen S., Liang P. (2020). Enhanced SO2 tolerance of FeCeOx/CNTs catalyst for NO and Hg0 removal by coating shell SiO2. Fuel Process. Technol..

[17] He C., Xu B.-T., Shi J.-W., Qiao N.-L., Hao Z., Zhao J.-L. (2015). Catalytic destruction of chlorobenzene over mesoporous ACeOx (A=Co, Cu, Fe, Mn, or Zr) composites prepared by inorganic metal precursor spontaneous precipitation. Fuel Process. Technol..

[18] Shafiee M.R.M., Sadeghian M., Kargar M. (2017). ZnFe2O4-Fe2O3-CeO2 composite nanopowder: Preparation, magnetic properties, and 4-chlorophenol removal characterizations. Ceram. Int..

[19] Ouyang J., Zhao Z., Subi S.L., Yang H. (2018). Degradation of Congo Red dye by a Fe2O3@ CeO2-ZrO2/Palygorskite composite catalyst: Synergetic effects of Fe2O3. J. Colloid Interface Sci..

[20] Shanmugam V., Sanjeevamuthu S., Jeyaperumal K.S., Vairamuthu R. (2019). Construction of α-Fe2O3/CeO2 decorated g-C3N4 nanosheets for magnetically separable efficient photocatalytic performance under visible light exposure and bacterial disinfection. Appl. Surf. Sci..

[21] Liu Y., Szeifert J.M., Feckl J.M., Mandlmeier B., Rathousky J., Hayden O., Fattakhova-Rohlfing D., Bein T. (2010). Niobium-Doped Titania Nanoparticles: Synthesis and Assembly into Mesoporous Films and Electrical Conductivity. ACS Nano.

[22] Kruefu V., Peterson E., Khantha C., Siriwong C., Phanichphant S., Carroll D.L. (2010). Flame-made niobium doped zinc oxide nanoparticles in bulk heterojunction solar cells. Appl. Phys. Lett..

[23] Yue J., Suchomski C., Voepel P., Ellinghaus R., Rohnke M., Leichtweiss T., Elm M.T., Smarsly B.M. (2017). Mesoporous niobium-doped titanium dioxide films from the assembly of crystalline nanoparticles: Study on the relationship between the band structure, conductivity and charge storage mechanism. J. Mater. Chem. A.

[24] Piggott E.K., Hope T.O., Crabbe B.W., Jalbert P.-M., Orlova G., Hallett-Tapley G.L. (2017). Exploiting the photocatalytic activity of gold nanoparticle-functionalized niobium oxide perovskites in nitroarene reductions. Catal. Sci. Technol..

[25] Zhang C., Ikeda M., Uchikoshi T., Li J.-G., Watanabe T., Ishigaki T. (2011). High-concentration niobium (V) doping into TiO2 nanoparticles synthesized by thermal plasma processing. J. Mater. Res..

[26] Kadyrzhanov K.K., Egizbek K., Kozlovskiy A.L., Zdorovets M. (2019). Synthesis and Properties of Ferrite-Based Nanoparticles. Nanomaterials.

[27] Gao S., Zhang W., Zhou H., Chen D. (2018). Magnetic composite Fe3O4/CeO2 for adsorption of azo dye. J. Rare Earth.

[28] Channei D., Inceesungvorn B., Wetchakun N., Phanichphant S. (2014). Synthesis of Fe3O4/SiO2/CeO2 core–shell magnetic and their application as photocatalyst. J. Nanosci. Nanotechnol..

[29] Gan G., Liu J., Zhu Z., Yang Z., Zhang C., Hou X. (2017). A novel magnetic nanoscaled Fe3O4/CeO2 composite prepared by oxidation-precipitation process and its application for degradation of orange G in aqueous solution as Fenton-like heterogeneous catalyst. Chemosphere.

[30] Egizbek K., Kozlovskiy A.L., Ludzik K., Zdorovets M.V., Ibragimova M.A., Marciniak B., Jazdzewska M., Chudoba D., Nazarova A., Kontek R. (2020). Application of Fe2O3/CeO2 nanocomposites for the purification of aqueous media. Appl. Phys. A-Mater..

